# LC-MS-MS quantitative analysis reveals the association between FTO and DNA methylation

**DOI:** 10.1371/journal.pone.0175849

**Published:** 2017-04-28

**Authors:** Yuting Zhu, Guangyu Zhou, Xuebin Yu, Qiang Xu, Kai Wang, Dan Xie, Qingkai Yang, Lina Wang

**Affiliations:** Department of Oncology, Second Affiliated Hospital, Institute of Cancer Stem Cell, DaLian Medical University, Dalian, Liaoning, China; National University of Singapore, SINGAPORE

## Abstract

Fat mass and obesity-associated protein (FTO) is α-ketoglutarate-dependent dioxygenase and responsible for demethylating N6-methyladenosine (m6A) in mRNA, 3-methylthymine (m3T) in single-stranded DNA (ssDNA) and 3-methyluracil (m3U) in single-stranded RNA (ssRNA). Its other function remains unknown but thousands of mammalian DNA show 5-methyl-2'-deoxycytidine (5mdC) modification and 5mdC demethylases are required for mammalian energy homeostasis and fertility. Here, we aimed to confirm whether FTO proteins can demethylate 5mdC in DNA. However, we found that FTO exhibits no potent demethylation activity against 5mdC in vitro and in vivo by using liquid chromatography-tandem mass spectrometry (LC-MS-MS). The result showed FTO demethylase has the characteristics of high substrates specificity and selectivity. In addition, we also used immunofluorescence technique to demonstrate overexpression of wild type TET2, but not FTO and mutant TET2 in Hela cells results in higher levels of 5-hydroxymethyl-2'-deoxycytidine (5hmdC) generated from 5mdC. In conclusion, our results not only reveal the enzymatic activity of FTO, but also may facilitate the future discovery of proteins involved in epigenetic modification function.

## Introduction

DNA methylation is an epigenetic modification, which does not change DNA sequence but plays important roles in a variety of cellular processes, including regulation of gene expression, genomic imprinting, X-chromosome inactivation, suppression of transposable elements, maintenance of epigenetic memory, and also important in cell differentiation [1–6]. Some studies reported DNA methylation, such as 5-methyl-2'-deoxycytidine (5mdC), always exists in promoter, which shows a correlation with gene expression or silencing [7]. DNA methylation is catalyzed by methyltransferase and removed by demethylase [8], and DNA methylation at 5 position of cytosine in the mammalian genome is a key epigenetic event and critical for various cellular processes [9].

While intensive efforts have been directed at investigating an active mechanism of DNA demethylation involving ten-eleven translocation (TET) methylcytosine dioxygenase and thymine DNA glycosylase (TDG) proteins [10], other potential demethylase towards 5mdC are less well known. TET genes encode a Fe (II) /αKG-dependent dioxygenases family and hydroxylate 5mdC to 5hmdC, which is then converted to unmodified cytosine through multiple mechanisms [7]. Importantly, all AlkB family proteins contain an analogous core catalytic domain called the double-stranded b-helix (DSBH) fold, which is conserved among Fe (II) / αKG-dependent dioxygenases. And the same fold also exists in TET family [2]. FTO is homologous to the DNA repair AlkB protein. The AlkB family members utilize Fe (II), a-KG and dioxygen to perform oxidative repair of alkylated nucleobases in DNA and RNA. So we speculate that these dioxygenases may catalyze demethylation of more than one methylated nucleic acid substrates.

Increasing evidences suggest that methylation modifications existing in RNA could be dynamic and even have regulatory roles similar to those of protein and DNA modifications [11]. Among these modifications, m6A is the most abundant modification in mammalian mRNA, while also existing in tRNA [12–15]. Many biological experiments have suggested that m6A in RNA analogous to 5mdC in DNA is dynamic and regulated by many enzymes, and m6A also involved in RNA processing and determined stability of the mammalian circadian clockwork [16–18]. FTO belongs to the Fe (II) and α-KG-dependent AlkB dioxygenase family, which was associated with body mass index, predisposes to childhood and adult obesity [19–21]. FTO was originally known as a demethylase to oxidatively remove m6A modification in mRNA [12]. Previous research has also shown FTO was able to catalyze oxidation of m3T in ssDNA [22]. These discoveries have assigned FTO as a nucleic acid demethylase that may work on DNA or RNA and also have raised a very interesting question: Is 5mdC in ssDNA or dsDNA a substrate for FTO protein?

To find out the functional roles of FTO in epigenetic modifications, we present here the study of biochemical activity of recombinant FTO protein in vitro and in vivo using liquid chromatography-tandem mass spectrometry (LC-MS-MS) and immunofluorescence technique. However, FTO as a demethylase exhibits no potent demethylation activity against 5mdC in vitro and in vivo. So, we confirm that FTO have drastically preference for demethylating m6A in RNA, instead of 5mdC in DNA. Moreover, we also found overexpression of wild type TET2, but not FTO and mutant TET2 in Hela cells results in higher levels of 5hmdC generated from 5mdC. Although our results demonstrated that FTO plays no obvious role in demethylating 5mdC in DNA, the research about FTO may contribute significantly to exposure key role of FTO in epigenetic regulation.

## Materials and methods

### Cloning, expression and purification of FTO

The human FTO gene was subcloned into a pET-28a vector to generate the plasmid pET-28a-FTO-6his. Then, the plasmid pET-28a-FTO-6his was overexpressed in BL21(DE3)-PlysS *E*. *coli*, which was grown in the nutrient-rich medium 32Y (containing 3.2% yeast extract, 0.8% peptone and 0.58% NaCl in 10 mM Tris-HCl, pH 7.4 at 30°C) [23]. Cells were induced with 0.1 mM isopropyl β-D-1-thiogalactopyranoside (IPTG) for 24 hrs at 20°C after cells reaching OD_600_ of 0.4~0.5. Induced BL21 (DE3)-PlysS host cells without any plasmid were used as negative control. Resultant cells were harvested by centrifugation at 6,000 g for 10 min and washed twice with cold phosphate-buffered saline (PBS). Collected cells were broken by ultrasonic wave and centrifugated at 2,4000 g and 4°C for 30 min to remove unbroken cells and debris. The soluble fraction was purified by using Ni sepharose 6 Fast flow (GE Healthcare). The supernatant containing FTO was loaded onto the column to maximize the binding of the protein to resin. The resins were washed five times with binding buffer (containing 10% glycerol and 20 mM imidazole, at pH 7.4 in PBS) to remove non-specifically bound proteins. The target protein was eluted by elution buffer (binding buffer containing 300 mM imidazole). Purified FTO was concentrated using an Amicon Ultrafree centrifugal filter (Millipore) with a cutoff of 10 kDa, which simultaneously removed imidazole from the protein solution. Proteins from relevant fractions were analyzed by SDS-PAGE and western blot. All of the protein purification procedures were performed at 4°C.

### In vitro FTO demethylation assay

The methylated nucleic acids (dsDNA, ssDNA and ssRNA) were incubated with purified FTO in 50 μl reaction buffer (10 mM Tris-HCl, 200 mM NaCl, 100 μM Fe^2+^, 200 μM α-KG, 1 mM Vc) at 37°C for 0 min, 15 min, 30 min, 1 hr, 2hrs and 4hrs. Purified FTO protein were used at 0, 0.01, 0.1, 0.5, 1, 2, 4 μg as indicated. After reaction, all samples were inactivated by adding 1 μl EDTA (0.5 M) and heated at 95°C for 5 min, following by adding 0.5 μl proteinase K (20 mg/ml) into the each reaction at 56°C for at least 15 min to completely digest the precipitated protein. And then the mixture was treated with 72°C for 15 min to inactivate the proteinase K. The reacted nucleic acid substrates (1 μg) were enzymatically digested into single nucleosides by mixture with 1 U DNase I or RNase A (NEB), 1 U nuclease P1 (Sigma) and 1 U alkaline phosphatase (Takara). And the completely digested DNA or RNA were subjected to LC-MS-MS analysis.

### Quantification of 5hmdC in DNA by LC-MS-MS

The expression plasmids encoding FLAG-FTO, FLAG-TET2 were transfected into HEK293 cells with Lipofectamine Reagent 2000 (Invitrogen). After 36 hrs, genome DNA was extracted with DNA purification Kit (Promega) following the manufacturer’s instruction. 5 μg of genomic DNA was completely digested into single nucleosides by 2 U DNase I (NEB) and 10 U DNA degradase plus (Zymo Research) in a total volume of 50 μL, and the mixture incubated at 37°C for 12 hrs. The solution was diluted 2 times, and 10 μl of the solution was subjected to LC-MS-MS analysis. The nucleosides were separated by reverse phase ultra-performance liquid chromatography on a C18 column, with mass spectrometry detection using AB SCIEX QTRAP 5500 LC-MS-MS in positive electrospray ionization mode. In brief, the digested DNA was performed using the mobile phase of 92% water (containing 0.1% formic acid) and 8.0% methanol at a flow rate of 400 μL/min. The ionspray voltage was maintained at 5500 V. The turbo gas temperature was set at 550°C. MRM was used to monitor analyte parent ion→product ion: m/z 228.0 to 112.0 (collision energy (CE) 15 V; declustering potential (DP) 60 V) for dC; m/z 242.1 to 126.0 (CE 13 V; DP 60 V) for 5mdC; m/z 258.0 to 142.0 (CE 22 V; DP 60 V) for 5hmdC. Both Q1 and Q3 quadrupoles were maintained at unit resolution. Analyst 1.6.1 software (Applied Biosystems, Darmstadt, Germany) was used for data acquisition and processing.

### Quantification of m6A in total RNA and mRNA by LC-MS-MS

The expression plasmid encoding FLAG-FTO was transfected into HEK293 (ATCC) cells with Lipofectamine Reagent 2000 (Invitrogen). After 36 hrs, total RNA was isolated using RNeasy Mini Kit (QIAGEN) following to the manufacturer’s instruction. Dynabeads Oligo (dT)_25_ (Thermo Fisher Scientific Inc) was used to purify the ployadenylated mRNA from total RNA. 1 μg of RNA (total RNA and mRNA) was digested by 2 U nuclease P1 (Sigma) in 40 μl buffer (10 mM Tris-HCl pH 7.0, 100 mM NaCl, 2.5 mM ZnCl_2_) at 37°C for 12 hrs, followed by incubating with 1 U alkaline phosphatase at 37°C for 2 hrs. RNA solution was diluted 10 times, and 10 μL of the solution was injected into LC-MS-MS. The nucleosides were separated by reverse phase high-performance liquid chromatography on an Agilent C 18 column (5-μm particle size, 150 mm x 2.1 mm), couple with mass spectrometry detection using AB SCIEX QTRAP 5500. The mass spectrometer was operated under positive ionization using MRM mode. The mobile phases (delivered at 0.40 ml/min) consisted of H_2_O for A and methanol for B. An isocratic elution (72% A and 28% B) was performed at stop time of 3 min. The MRM transitions were monitored as follows: m/z 267.9 to 136.1 for A (collision energy (CE): 25 V; declustering potential (DP): 10 V; Entrance potential (EP): 10 V; collision cell exit potential (CXP): 13 V); m/z 282.1 to 150.1 for m6A (CE 30 V; DP 12 V, EP 8V; CXP 10V). The following instrument parameters were used: column temperature: 25°C; ionspray voltage: 5500 V; turbo gas temperature: 550°C. Both Q1 and Q3 quadrupoles were maintained at unit resolution. Analyst 1.6.1 software was used for peak areas quantification and data processing.

### Immunofluorescence assay

Hela cells (ATCC) were plated onto glass coverslips and the expression plasmids encoding FLAG-TET2 domain, FLAG-TET2 mutant domain (H1304Y, D1306A), and FLAG-FTO were transfected into Hela cells with Lipofectamine Reagent 2000 (Invitrogen). The immunofluorescence method was same as previously described [24] or with a minor modification. 36 hrs post-transfection, cells were first fixed by 4% paraformaldehyde for 10 min. The cells were then washed with cold PBS and treated with 0.2% Triton-100 in PBS for 10 min to permeate cells. Permeabilized cells were then washed and incubated for 1 hr with blocking buffer (2% BSA in PBS buffer) to block non-specific protein-protein interactions. For 5hmdC staining, the permeabilized cells were denatured with 2 N HCl for 15 min, neutralized with 100 mM Tris-HCl (pH 8.5) for 15 min before blocking [25]. After washing with PBS, the cells were incubated for 1 hr with anti-5hmdC antibody (sigma) from rabbit. This was washed three times with PBS and followed by 1 hr incubation with Alexa Fluor 488 goat anti-rabbit IgG (Invitrogen). After washing, cells were incubated with anti-FLAG antibody (sigma) from mouse for 1 hr. This was washed three times with PBS and followed by 1 hr incubation with Alexa Fluor 568 goat anti-mouse IgG (Invitrogen). After three consecutive 5 min washes with PBS, coverslips were mounted with a drop of mounting medium supplemented with 4,6-diamidino-2-phenylindole dihydrochloride (DAPI). Images were acquired using Leica laser confocal microscopy.

## Result

### No significant oxidation of 5mdC catalyzed by FTO in vitro

**Fig 1:**
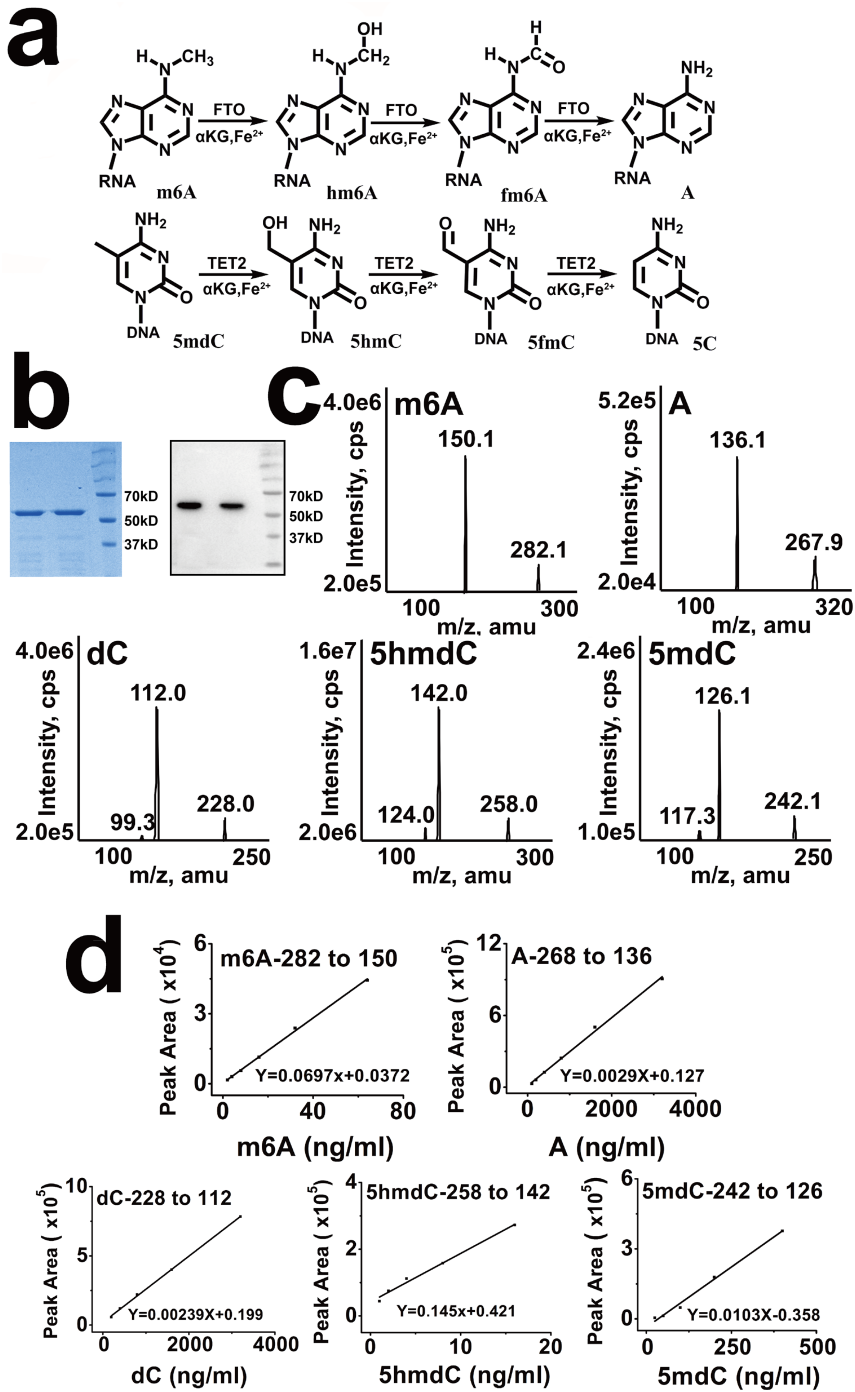
Characterization of several kinds of nucleoside modification in DNA and RNA. (a) Proposed oxidative demethylation of m6A to N6-hydroxymethyladenosine (hm6A) and N6-formyladenosine (f6A) in RNA by FTO and oxidation of 5mdC to 5hmdC and 5-formylcytosine (5fC) in DNA by TET2. (b) Coomassie staining and western blot of His-tagged full-length human FTO proteins purified from BL21(DE3)-PlysS *E*. *coli*. (c) Base ion mass transitions for LC-MS-MS analysis of A, m6A, dC, 5mdC and 5hmdC standard. The MRM transitions were monitored as follows: 267.9 to 136.1 (A); 282.1 to 150.1 (m6A); 228.0 to 112.0 (dC); 258.0 to 142.0 (5hmdC); 242.1 to 126.1 (5mdC). (d) LC-MS-MS standards curves of A, m6A, dC, 5mdC and 5hmdC.

**Fig 2:**
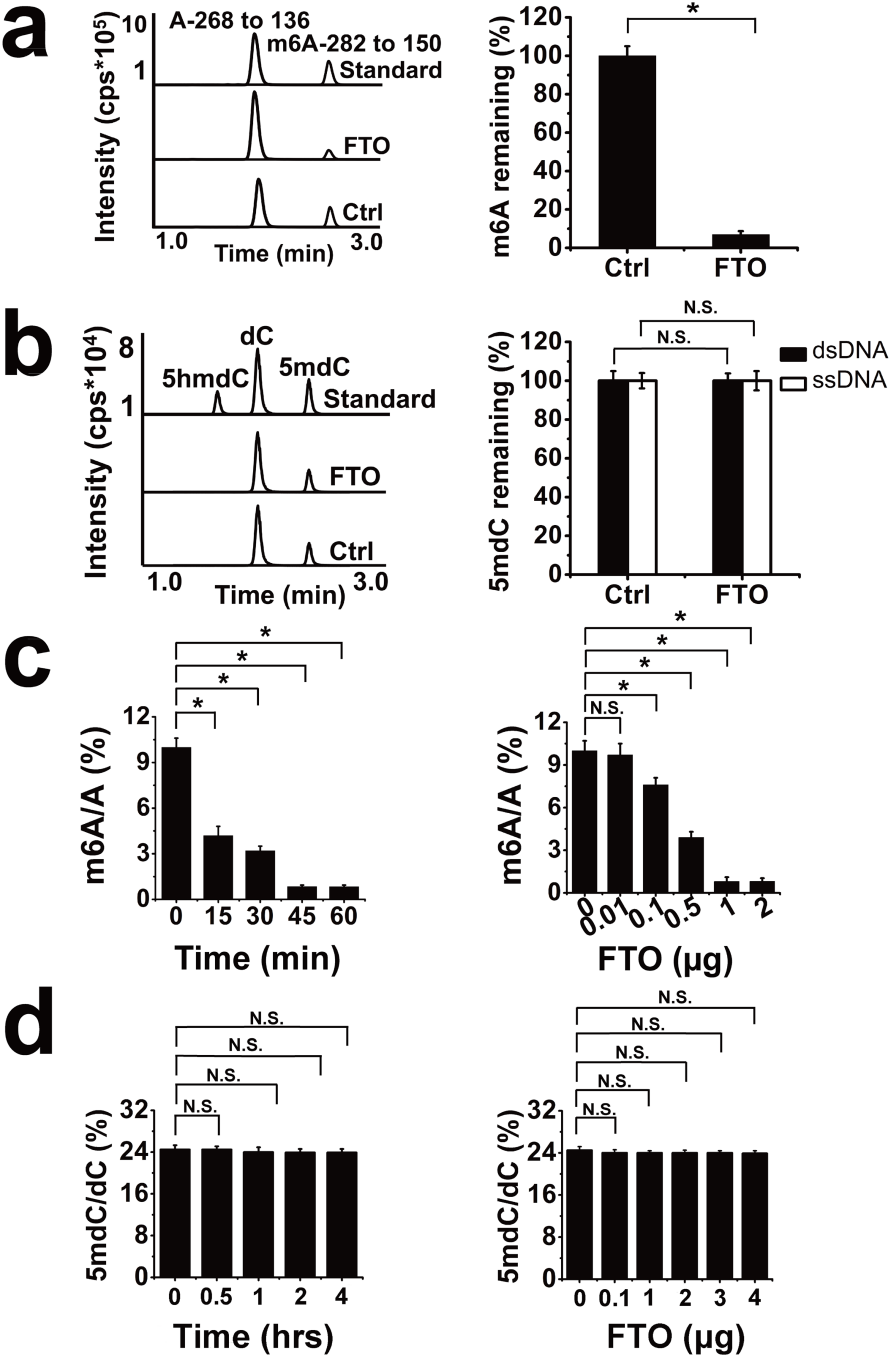
LC-MS-MS assay measures the demethylation activity of FTO in vitro. (a) (left) LC-MS-MS profiles of nucleosides derived from enzymatic hydrolysis of RNA samples containing m6A upon treatment with the FTO protein. The upper LC-MS-MS profile shows nucleoside A and m6A standards. (Right) Quantitation of m6A in vitro reaction system of FTO and synthetic RNA substrates. (b) (left) LC-MS-MS profiles of nucleosides derived from enzymatic hydrolysis of DNA samples containing 5mdC upon treatment with the FTO proteins. The upper LC-MS-MS profile shows nucleoside 5hmdC, dC and 5mdC standards. (Right) Quantitation of 5mdC in vitro reaction system of FTO and synthetic DNA. (c) FTO catalyzes the demethylation of m6A in a dose- and time-dependent manner. (d) FTO shows no obvious converting 5mdC to 5hmdC in DNA with the increase of the dose of FTO and reaction time. *p < 0.05; N.S.: No Significance. Error bars, mean ± S.E.M. for triplicate experiments.

As shown in Fig 1a, FTO and TET2 oxidatively demethylated m6A in RNA and 5mdC in DNA, respectively [7, 26]. Therefore, we ask if FTO can oxidize 5mdC to 5hmdC in DNA. Purified FTO protein was determined using SDS-PAGE followed by Coomassie blue staining and western blot (Fig 1b). To validate the idea, multiple reaction monitoring (MRM) was used to determine base ion mass transitions of m6A (282.1 to 150.1), A (267.9 to 136.1), dC (228.0 to 112.0), 5hmdC (258.0 to 142.0), and 5mdC (242.1 to 126.1) (Fig 1c). Standard curves were built to quantify m6A, A, dC, 5hmdC, and 5mdC modification (Fig 1d). First, we test the demethylation activity of recombinant human FTO protein for ssRNA substrates with site-specifically incorporated m6A (5’-AUUGUCA(m6A)CAGCAGC-3’). This demethylation activity assay was performed as described in Methods. Fortunately, the result of mass spectrum analysis showed that m6A in 1 μg of ssRNA has been almost completely demethylated by 1 μg of FTO (Fig 2a).

Then, ssDNA and dsDNA with site-specifically incorporated 5mdC (5’-ATTGTAG(5mdC)CAGCAGA-3’) were selected as substrates at the same condition to test demethylation activity of FTO in vitro. However, no detectable 5hmdC generated from 5mdC was quantified using LC-MS-MS (Fig 2b). In addition, we further determined whether FTO has dose-and time-dependent enzymatic activity in a constant substrate concentration (1 μg). As shown in Fig 2c, FTO significantly reduced m6A modification in a dose- and time-dependent manner. But that, unfortunately, for 5mdC, FTO showed no obvious converting 5mdC to 5hmdC in ssDNA with the increase of reaction time and the dose of FTO (Fig 2d), as well as in dsDNA (data not shown). We determined detailed reaction kinetics of FTO on m6A-containing ssRNA and 5mdC-containing ssDNA at 37°C (S1 Fig and Table 1). Taking these results together, we reveal a meaningful phenomenon that FTO showed strong preference to remove m6A in RNA instead of 5mdC in DNA and only a few specific DNA as substrates could be demethylated by FTO, such as m3T in ssDNA [22].

**Table 1:** Kinetic constants for FTO demethylation of m6A in ssRNA and 5mdC in DNA.

Substrate	K_m_ (μM)	K_cat_ (S^-^)	K_cat_/ K_m_ (S^-^ μM^-^)
ssRNA (m6A)	0.383±0.34	0.00289±0.005	0.00755
ssDNA(5mdC)	−	−	−
dsDNA(5mdC)	−	−	−

#### No detectable demethylation of 5mdC in genome DNA catalyzed by FTO in vivo

**Fig 3:**
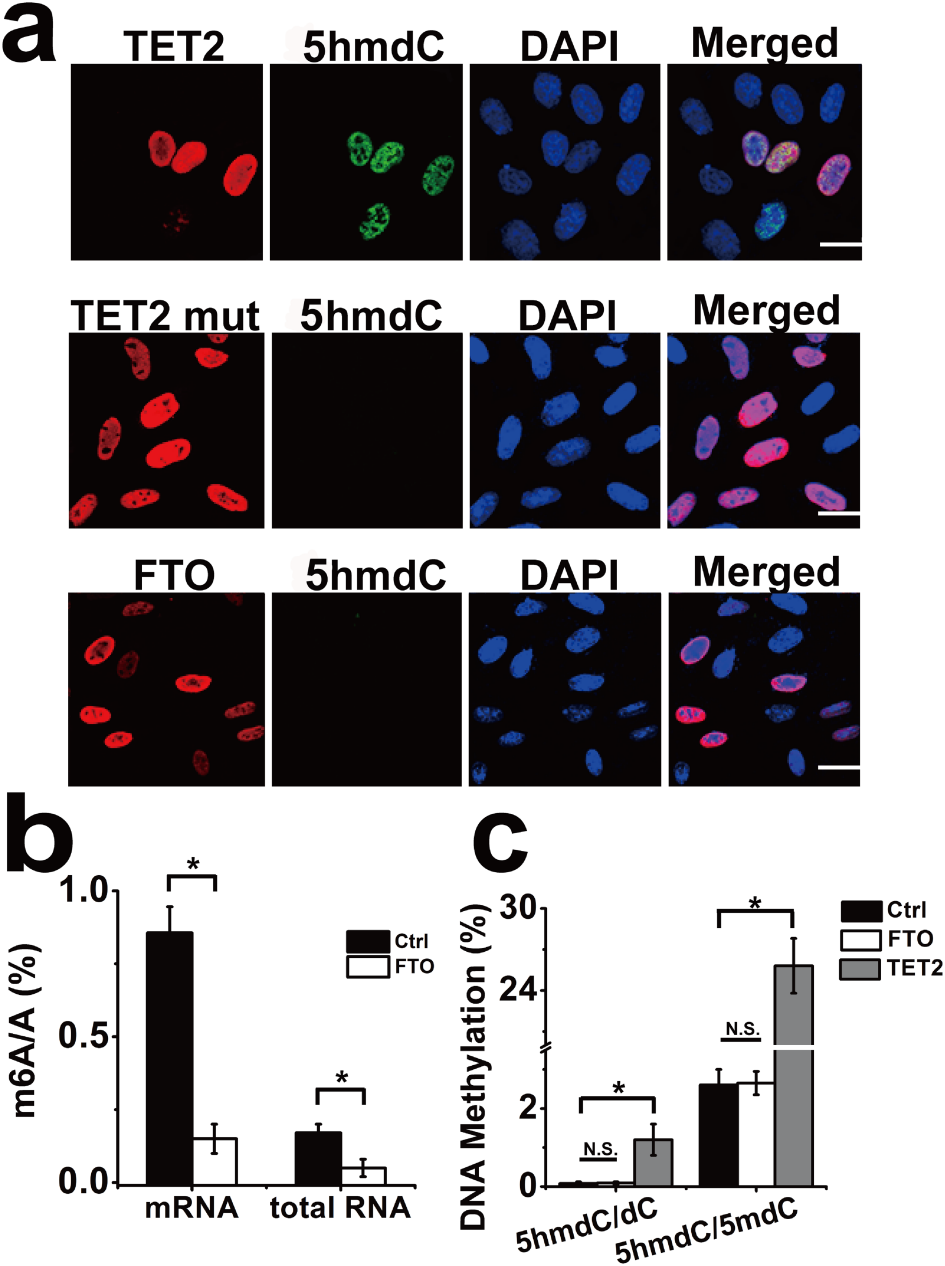
Immunofluorescence and LC-MS-MS experiments measure the demethylation activity of FTO in vivo. (a) Immunofluorescence analysis of 5hmdC level generated from 5mdC in FTO or TET2 overexpressed Hela cells. Cells were stained with anti-5hmdC antibody (green), showed that 5hmdC signal is obvious in the TET2 overpexpressed cells, instead of TET2 mutant or FTO gene transfected cells. Nuclei are stained by DAPI. Scale bar: 0–50 μm. (b) LC-MS-MS quantification analysis showed percentage of m6A/A in mRNA and total RNA isolated from control and FTO overexpressed cells. (c) LC-MS-MS quantification analysis showed percentage of 5hmdC/dC, 5hmdC/5mdC in DNA isolated from control, FTO and TET2 overexpressed cells. *p < 0.05; N.S.: No Significance. Error bars, mean ± S.E.M. for triplicate experiments.

5mdC is a major epigenetic modification of mammalian DNA [27]. A demethylase TET2, commonly existing in mammalian cell, can convert 5mdC to 5hmdC in DNA [28]. Similarly, FTO also as a mammalian demethylase, was reported that responsible for ssRNA and ssDNA demethylation. Given that both FTO and TET2 are demethylase of nucleic acid, we wonder if FTO possess the same function like TET2 to regulate 5mdC demethylation in mammalian cells. To explore this issue, we performed the immunofluorescence experiment. In this assay, overexpression of wide type TET2 and mutant TET2 were used as positive and negative control, respectively. As shown in Fig 3a, a striking 5hmdC staining signal was observed in the nucleus when wild type TET2 gene was overexpressed in Hela cells. In contrast, 5hmdC signal was extremely weak in FTO and mutant TET2 overexpressed cells. Thus, immunofluorescence experiment confirmed that overexpression of FTO led to undetectable level of 5hmdC in nuclear DNA.

To further determine the biological role of FTO in DNA demethylation, we employed LC-MS-MS to measure the abundance of 5hmdC in FTO-transfected HEK293 cells. The LC-MS-MS assays showed that, after FTO overexpressing about 48 hrs, ~40% and ~60% decrease of the m6A level in total RNA and in mRNA were consistently observed in repeated experiments (Fig 3b). But, compared with the control, no significant increasing of 5hmdC, generated from 5mdC catalyzed by FTO, was detected in DNA (Fig 3c). Indeed, HEK293 cells transfected with TET2 increased the level of 5hmdC by 10-fold compared to the control and FTO group. These experiments in vivo further supported that FTO had no obvious effect on oxidization of 5mdC in genome DNA.

## Discussion

In recent years, more and more researchers found both DNA and RNA methylation can be reversed by multiple dioxygenase, which opens a door to further understand mechanism of DNA or RNA methylation and demethylation [2]. To date, examining of FTO as a biologically relevant demethylase have mainly been limited to oxidation of RNA and ssDNA. The prevailing view is that FTO catalyzes oxidation of m6A in RNA and m3T in ssDNA [22, 29]. Moreover, 5mdC, as the epigenetic marker of DNA, exerts a predominant role in mammalian genome [30]. And the important function of 5mdC modification in mammals has attracted much interest in the field of the epigenetic control. So we hypothesized whether there is another physiological demethylase, such as FTO, responsible for converting 5mdC to 5hmdC in dsDNA and ssDNA.

In this article, firstly, it was showed that FTO demethylation activity towards 5mdC in dsDNA and ssDNA was tested by LC-MS-MS and immunofluorescence experiments. The synthetic methylated DNA substrates were not significantly oxidized in the presence of FTO, and also were not significantly demethylated with the increase of reaction time and dose of FTO in vitro (Fig 2d). Nevertheless, we wonder if the decrease of 5mdC level was caused by FTO in vivo. 5hmdC as an intermediate product, was produced while TET2 protein catalyzed the demethylation of 5mdC in genome DNA. But the mutant TET2 in this article does not have this kind of function. Hence, the immunofluorescence experiment was implemented and exhibited that excess wild type TET2 instead of FTO caused apparent increasing of cellular 5hmdC levels (Fig 3a). Additionally, we also quantified the abundance of 5mdC, 5hmdC, dC from genome DNA. The results revealed that levels of 5mdC, 5hmdC and dC had no detectable changes, while FTO was overexpressed (Fig 3c). Considering the negligible repair of ssDNA and dsDNA substrates with 5mdC by FTO protein, indicating that the processivity of oxidizing 5mdC to 5hmdC may be regulated by substrate preference and/or the accessibility of TET dioxygenases instead of FTO [31].

Although, LC-MS-MS and immunofluorescence experiments suggested FTO had no obvious effect on oxidation of 5mdC in DNA, the study that searching a new demethylase for ssDNA or dsDNA with 5mdC modification substrates has provided a new perspective on how heritable DNA methylation pattern may be dynamically regulated in the mammalian genome. We will be more committed to find more regulation of 5mdC modification. On the other hand, we also aim to find out biological effect of FTO in epigenetic modifications. And these findings will bring an insight into understanding the potential function of FTO in epigenetics and gene expression in higher eukaryotes. We believe that further insight into the functional properties of FTO not only reveals complicated clinical phenomenon, but also may bring expectation to heal many human disease.

## Supporting information

S1 Fig Michaelis-Menten’s plot of the steady-state kinetics of FTO demethylating m6A in ssRNA and 5mdC in DNA. (TIF)
