# Supplementary figures and images for: LC-MS-MS quantitative analysis reveals the association between FTO and DNA methylation
Source: PLoS One. 2017 Apr 28;12(4):e0175849. doi: 10.1371/journal.pone.0175849 (PMC5409144; doi:10.1371/journal.pone.0175849)

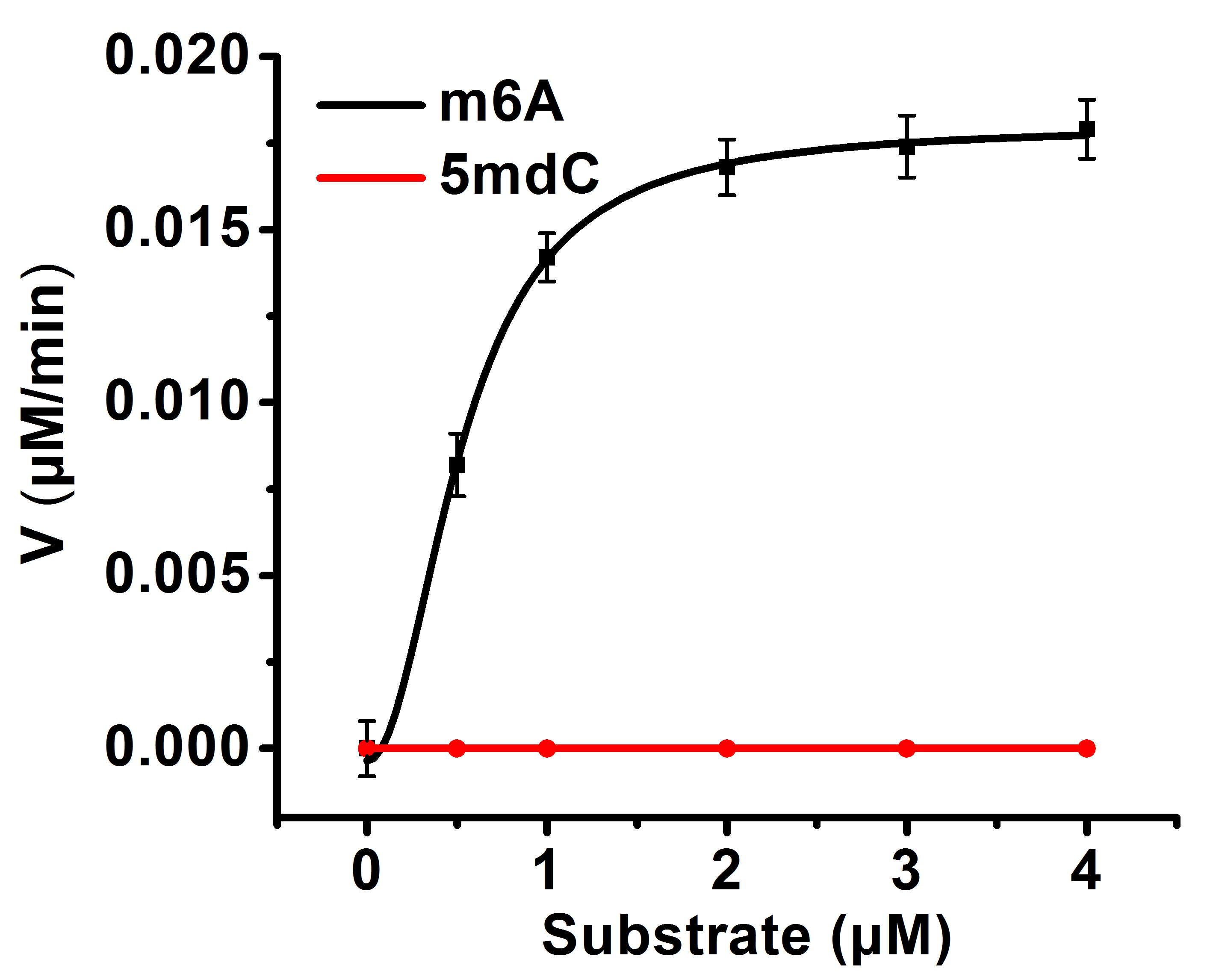

Supplement: S1 Fig — (TIF) [file pone.0175849.s001.tif]
